# Magnetization transfer MRI of third trimester fetal brain: a pilot of gestational myelin imaging

**DOI:** 10.3389/fped.2025.1443387

**Published:** 2025-05-09

**Authors:** Siddharth Sadanand, Rob Stobbe, Tim van Mieghem, Shiri Shinar, Pradeep Krishnan, Elka Miller, Greg Stanisz, Dafna Sussman

**Affiliations:** ^1^ Maternal Fetal Imaging Laboratory (MFI Lab), Institute for Biomedical Engineering, Science and Technology (iBEST), Departments of Biomedical Physics & Engineering, Toronto Metropolitan University, Toronto, ON, Canada; ^2^ Peter S. Allen MR Research Centre, Biomedical Engineering Department, University of Alberta, Edmonton, AB, Canada; ^3^ Fetal Medicine Unit, Department of Obstetrics and Gynaecology, Mount Sinai Hospital, Toronto, ON, Canada; ^4^ Department of Obstetrics & Gynaecology, University of Toronto, Toronto, ON, Canada; ^5^ Neuroradiology, Department of Diagnostic Imaging, The Hospital for Sick Children, Toronto, ON, Canada; ^6^ Department of Medical Biophysics, University of Toronto, Toronto, ON, Canada; ^7^ Physical Sciences, Sunnybrook Research Institute, Toronto, ON, Canada

**Keywords:** magnetic resonance, fetal imaging, brain imaging, myelin imaging, magnetization transfer, pulse sequence development

## Abstract

**Background:**

Magnetic resonance imaging (MRI) is commonly used as a tool for confirming a fetal brain abnormality suspected on ultrasound. Common clinical MRI sequences typically only reveal changes in the brain once there are gross abnormalities. Detection of more minute changes in brain tissue microstructure could permit earlier detection of complications, allowing for potentially more timely, effective interventions. Tissue microstructure corresponding to neuronal development can be captured before the appearance of broad anatomical changes using more advanced imaging, such as magnetization transfer (MT) MRI. This study aimed to investigate the feasibility of an MT MRI pulse sequence developed by the researcher, yarnball (YB) MT, for fetal brain imaging.

**Methods:**

A yarnball (YB) readout trajectory was used to accelerate imaging and increase sensitivity. A multiband saturation pulse was implemented to increase MT specificity from saturation transfer (ST) confounds. MT-weighted images were derived from three-point magnetization transfer ratio asymmetry (MTR_asym_) to reduce acquisition time to within a breath hold. Sensitivity and specificity were evaluated on agar phantoms with varied MT and ST confound concentrations. Pilot imaging was done with singleton third trimester gestations complicated with mild ventriculomegaly recruited from Mount Sinai Hospital.

**Results:**

YB MT covers a 350 mm 3D field of view (FoV) within a 13 s breath hold and a 28 s acquisition. The sequence demonstrated a limit of quantification (LOQ) of agar of 0.62% w/w and no dependence on glucose in agar phantoms with glucose ST confound. Pilot imaging *in vivo* of third trimester pregnancies with mild ventriculomegaly with the sequence revealed MT contrast in the fetal brain that was spatially consistent with the development of white matter at this gestational age. All participants reported the sequence and the breath hold to be tolerable.

**Conclusion:**

The developed YB MT pulse sequence is sensitive to fetal physiological MT signal is tolerable to participants, and does not demonstrate sensitivity to ST confounds in phantom imaging. While the breath hold was reported to be tolerable, motion artefacts and spiral trajectory blurring affected subjects' imaging. Ongoing work, including online reconstruction, expedited trajectories, and improvements in the signal-to-noise ratio should address these challenges. This proof of principle is a step towards the clinical translation of gestational metabolic imaging, such as MT imaging of fetal myelin, for the early detection of gestational complications.

## 1 Introduction

Fetal brain magnetic resonance imaging (MRI) plays a pivotal role in high-risk pregnancy, offering invaluable insights into prenatal brain development and aiding in the detection of potential abnormalities (1–6). The ability to identify complications at an early stage is paramount, as it allows for accurate counselling based on which parents can make decisions about pregnancy management and, in some cases, perinatal intervention ultimately leading to improved neonatal health outcomes (1, 7–9). In current clinical practice, fetal structural MRI is commonly used to investigate abnormalities detected on fetal neurosonogram, the appearance of which occurs well after the underlying pathology arises (10–12).

Microstructural imaging techniques hold great promise for enhancing our understanding of fetal brain development and early detection of pathology (10–12). Unlike conventional structural MRI, which primarily detects gross anatomical changes, microstructural imaging offers the advantage of probing tissue function and composition (13). By assessing cellular processes that give rise to microstructure, these advanced imaging techniques can provide a more comprehensive understanding of fetal brain health and could better inform clinical counselling on pregnancy management and guide targeted perinatal interventions (10–13). For instance, early detection could allow for expedited administration of maternal corticosteroids or delayed cord clamping to reduce the risk of intraventricular haemorrhage and promote white matter development (14, 15).

In this context, saturation transfer (ST) imaging has emerged as a valuable tool for fetal metabolic imaging, offering the potential to assess tissue function before the onset of structural changes (13). By selectively saturating specific metabolite chemical shifts, ST imaging allows for the quantification of metabolic processes with high sensitivity and specificity (16). The saturation that is transferred to the water pool accumulates across many exchanges over an exchange time, amplifying the metabolite signal as a function of exchange rate, exchange time, magnetization recovery, and saturation power and duration. This metabolite signal amplification ameliorates sequence sensitivity, mitigating the need for large voxels or multiple averages. Differing transfer mechanisms further allow contrast to be weighted by the exchange rate and spectral linewidth by adjusting the saturation pulse scheme accordingly (16). Magnetization transfer (MT) is one such mechanism in which contrast is derived from semisolid macromolecules and so could enable myelin imaging in the developing fetal brain (10–12, 16).

The translation of ST metabolic imaging techniques to the fetal brain presents several advantages alongside several challenges (10–12). One advantage ST imaging enables is higher metabolite sensitivity than other metabolic MRI such as MR spectroscopy (16). Another advantage ST imaging offers due to this higher metabolite signal is reduced imaging time (16). Despite these benefits to imaging the fetal brain, acquisition time remains a challenge due to imaging in a gestational setting, alongside maternal anatomy; maternal respiratory, cardiac, peristaltic, and spontaneous fetal motion have so far precluded the relatively long acquisition times of clinically translated adult metabolic MRI (10–12). An additional challenge in ST imaging is that the metabolic MT signal of interest can be obscured by confounding ST mechanisms, such as chemical exchange saturation transfer (CEST), direct saturation (DS, spillover), and the nuclear Overhauser effect (NOE) (16). Low fetal metabolite concentration and gestational anatomy also pose challenges for imaging (10–12), including coil distance, higher specific absorption rate (SAR) of radiofrequency (RF) energy, and dielectric signal dropout.

In this study, we aimed to investigate the feasibility of an MT MRI pulse sequence, termed yarnball (YB) MT, for fetal brain and myelin imaging. The pulse sequence was designed to address many of the challenges of fetal metabolic imaging, with a single breath hold 3D whole-uterus acquisition, and a saturation block implemented for sensitivity to MT and to be robust to ST confounds. Through a combination of phantom experiments and a pilot imaging study in third trimester gestations, we sought to evaluate the sensitivity, specificity, and clinical potential of the YB MT sequence for early detection of gestational complications affecting fetal brain development. Through this research, we aim to advance our understanding of fetal brain metabolism and pave the way for improved prenatal diagnosis and intervention strategies.

## 2 Materials and methods

### 2.1 Pulse sequence development

**Figure 1:**
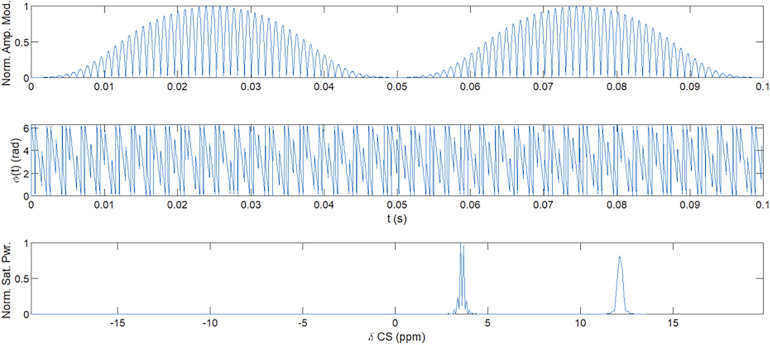
Multiband ZAPI saturation pulse. Amplitude (top) and phase (middle) modulation of the oscillator were used to obtain multiband saturation (bottom) at 12 ppm and 3.5 ppm. Multiband saturation (19) and ZAPI (18) reduce DS and CEST confounds for MT signal measurement.

The YB MT pulse sequence was developed using the stock *a_gre* Siemens sequence (17), for use on Siemens 3 T clinical and research scanners running software VE11C and XA30A. This *a_gre* sequence was modified to include arbitrary pre-saturation pulses for magnetization preparation and arbitrary readout trajectories (17) from text files. The saturation pulse used for imaging was a Fermi-apodised, single-lobed sinc pulse of 99.8 ms and flip angle (FA) = 2,000°. *Z*-spectrum alternating phase irradiation (ZAPI) saturation was used to mitigate DS (18). These saturation parameters were determined by the scanner continuous wave (CW) RF power amplifier (RFPA) limit and a conservative estimate of SAR limit (<0.8 W/kg, *B*_1_ = 0.72 µT). The multiband pulse saturated at both 12 ppm and 3.5 ppm as shown in Figure 1 to suppress ST confounds (19), and the saturation block consisted of 30 incoherent pulses 99.8 ms long with 1 ms pulse spacing and gradient spoiling. An earlier implementation of the multiband pulse was used for Participant 1 with saturation at 12, 2.88, 2.08, 1.28, and 0.66 ppm. Saturation pulse apodization, per-band saturation power, number of pulses, and duty cycle were determined through genetic algorithm optimization for MT signal.

**Figure 2:**
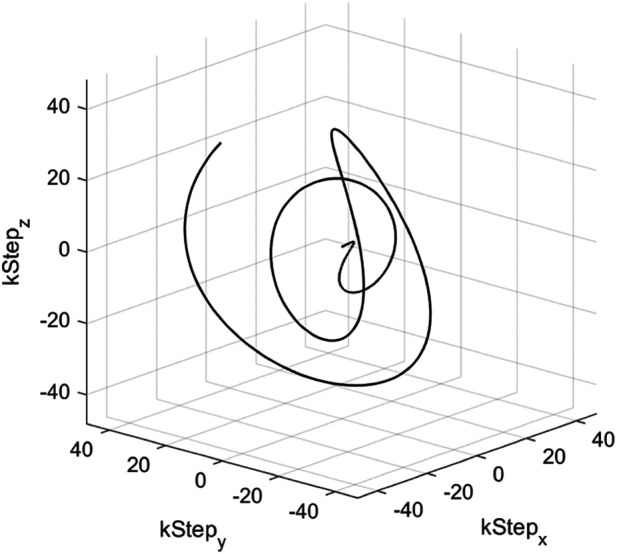
Depiction of a single yarnball interleave, TE = 0.12 ms, *T*_RO_ = 2 ms, TR = 3.3 ms used in SR trajectory.

The standard resolution (SR) acquisition trajectory was a gradient- and RF-spoiled steady-state free precession (SSFP) interleaved 3D yarnball (17) covering a 350 mm field of view (FoV) at 5 mm isotropic resolution. The trajectory constituted of 512 gradient and RF-spoiled ultrashort echo time (UTE) interleaves shown in Figure 2 with 2 ms readout time (*T*_RO_), echo time (TE) = 0.12 ms, repetition time (TR) = 3.3 ms, and FA = 3.15° from the Ernst angle for an expected phantom and fetal tissue-average $T_{1}\;\;\approx \;\;2.2\;\;s$ drawn from phantom relaxometry.

**Figure 3:**
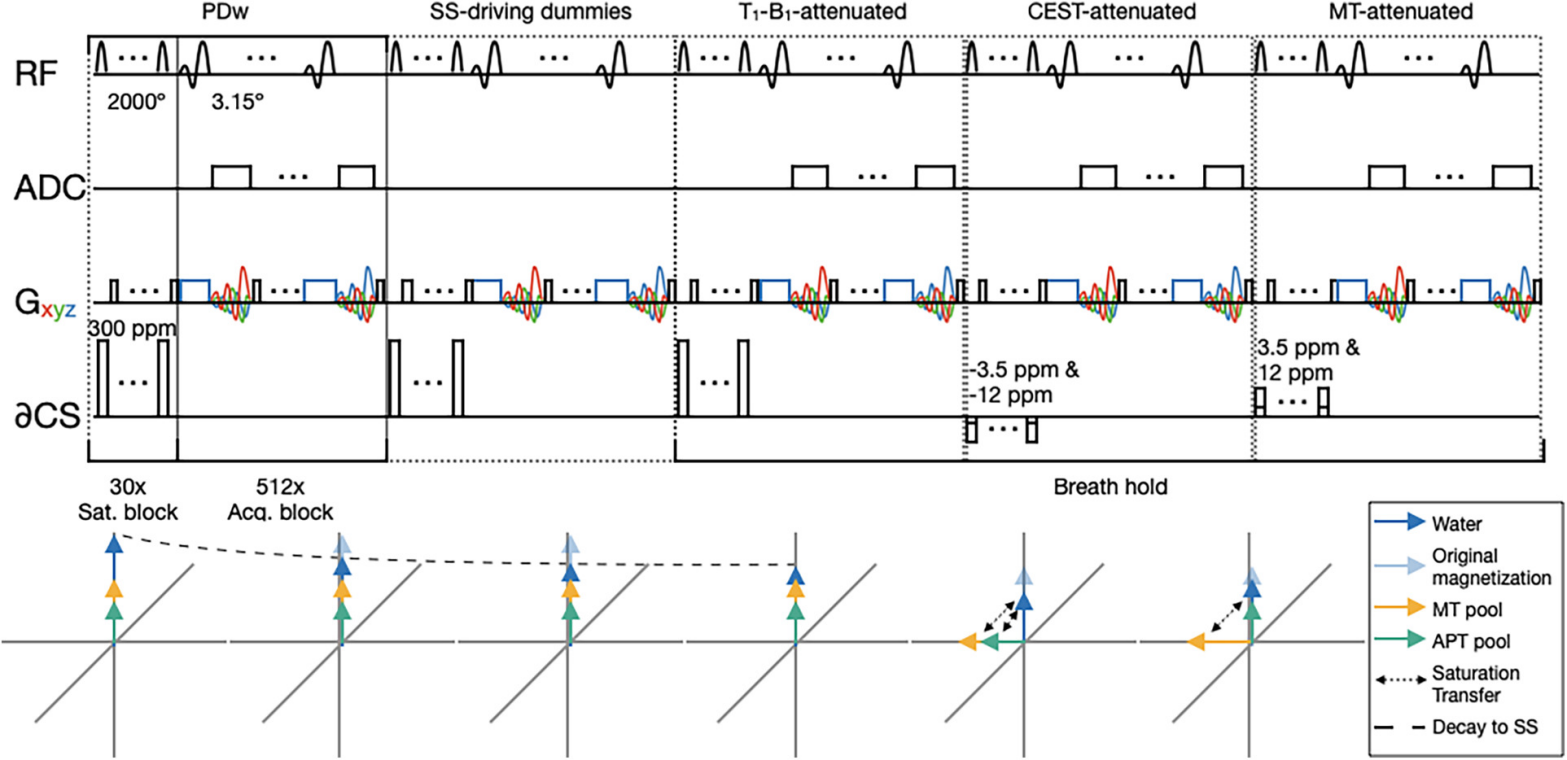
A pulse sequence diagram illustrating YB MT. Below is a depiction of the spin of three proton pools of interest over the course of YB MT; water is shown in blue, MT protons are shown in yellow, and CEST protons are shown in teal. Saturation transfer is shown with dashed arrows. DS is omitted here because ZAPI saturation effectively suppresses the saturation of long *T*_2_ components such as water (18). NOE is omitted here because multiband saturation effectively suppresses the saturation of pools with line widths narrower than the spacing between bands (19). The sequence is composed of five imaging blocks with contrast in the following order: PDw, SS-driving dummy acquisitions (image data not recorded), PDw *T*_1_- and *B*_1_-attenuated by the SS-driving dummies, CEST-attenuated, and MT-attenuated. A dashed line in the below spin plots shows how the water pool reaches a steady state over the course of the PDw and SS-driving dummy acquisitions, with original magnetization shown by the faded arrow. Acquisition blocks are gradient- and RF-spoiled SSFP that are consistent across imaging blocks. Saturation blocks vary the contrast between imaging blocks, consisting of *n* = 30, DC = 99%, *t*_sat_ per pulse = 99.8 ms, FA per pulse = 2,000°. Gradient traces are coloured such that *G_x_* is red, *G_y_* is green, and *G_z_* is blue. Where *G_x_*, *G_y_*, and *G_z_* are used together for spoiling, the *G_xyz_* trace is shown in black.

The sequence constituted a proton density-weighted (PDw) acquisition, a steady-state (SS)-driving dummy acquisition, a *T*_1_–*B*_1_-attenuated PDw acquisition, and MT- and CEST-attenuated acquisitions with 4 s magnetization recovery periods for a total acquisition time of TA = 27.5 s. An illustrative pulse sequence diagram is shown in Figure 3. A higher-resolution (HR) 150 mm FoV, 2 mm isotropic resolution, 578-interleave trajectory was developed and used for phantom calibration, but dielectric artefacts diminished signal-to-noise ratio (SNR) *in vivo* significantly.

### 2.2 Phantom preparation

**Figure 4:**
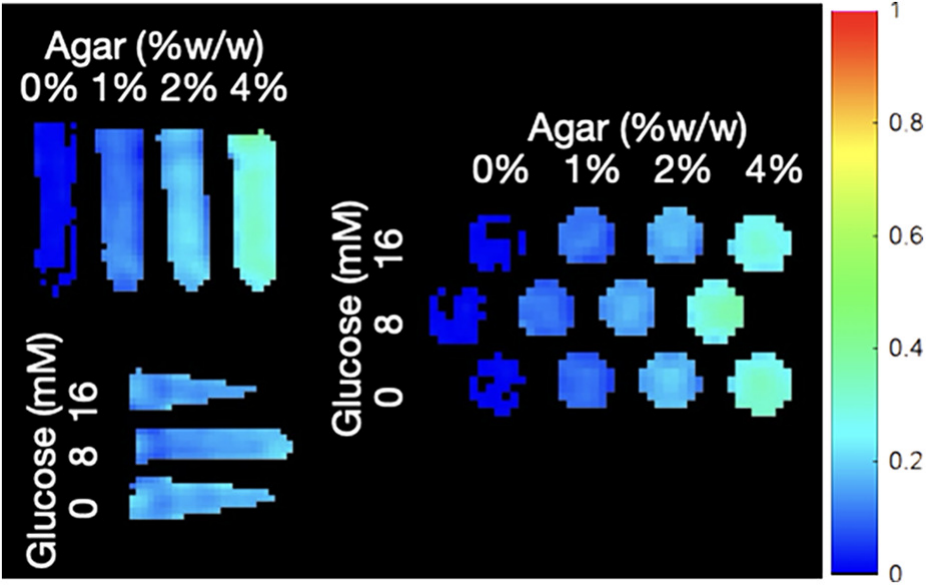
YB MTw image of the ST phantoms annotated with their agar MT and glucose CEST confound contents. The SR trajectory was used here.

An array of 12 phantoms with varying MT signal and chemical exchange ST that confounds MT measurements were produced to determine YB MT sensitivity to physiological MT and robustness to ST confounds. Phantoms were produced in deionized (DI) water (ρ=10MΩ·cm). D-(+)-glucose (99%, anhydrous, Alfa Aesar A16828) was added as an ST confound to produce a 16 mM solution. Glucose was chosen as an ST confound because its concentration in adult tissues is well characterized and maternal blood glucose is correlated to some aetiologies of growth restriction and gestational complications. A CEST confound of 8 mM was obtained by dilution of the 16 mM stock. To produce an MT signal, 0, 1, 2, or 4% w/w agar (Alfa Aesar A10752.36) was added to each respective array of glucose solutions, the agar was hydrated, and the solutions were heated in a double-boiling apparatus with stirring and watch glass until fully dissolved at $T\;\approx 100^{\circ }C$. Stirring was reduced to allow air bubbles to rise and then poured hot into 50 ml conical bottom centrifuge tubes. After cooling to ambient temperature, phantoms were stored continuously at 4°C. Imaging was conducted 4 days after phantom preparation (Figure 4). For 4 h prior to imaging, phantoms were allowed to equilibrate with ambient temperature.

### 2.3 Participant imaging

Five participants were recruited from the maternal–fetal medicine (MFM) neuroimaging clinic at Mount Sinai Hospital (MSH). Participant demographics and fetal outcomes are summarized in Table 1. Inclusion criteria were early-to-mid third trimester singlet pregnancies with a mild complication on fetal neurosonogram necessitating follow-up investigation by MRI. The exclusion criteria were maternal metabolic disorders, severe structural fetal neurodevelopmental complications on sonography beyond mild ventriculomegaly, fetal genetic abnormalities or aneuploidies, and maternal metabolism-modifying medication. All cases were reviewed by an MFM specialist for eligibility and were referred for clinical MRI to further investigate the finding of mild ventriculomegaly on detailed neurosonography. Participants were briefed on the study purpose, design, risks, and benefits of participation, and personal health information (PHI) confidentiality practices before being sent home with study information and consent form material for review, in accordance with the Hospital for Sick Children (SickKids) REB #1000073206 and MSH REB#19-0207-E. Apart from one participant whose imaging was split across clinical and research scanners, participants were booked for combined clinical and research protocol scans on a Siemens 3 T Magnetom Prisma Fit (Siemens Healthineers AG, Erlangen, Germany) research scanner at SickKids. A 32-channel spine bed coil and 18-channel Siemens body flex coil were used for imaging, with the body flex coil placed with an inferior edge aligned to the participant's iliac crest to reduce signal dropoff. Participants had fasted for 4 h prior to their imaging appointment and were instructed through a 13 s breath hold for the duration of the latter three acquisitions in the sequence. Dual flip angle *B*_1_, dual echo time B_0_ phase map, inversion–recovery (IR) *T*_1_, and multiecho SE *T*_2_ maps were also acquired, alongside diffusion tensor imaging (DTI) and resting state functional MRI (rs-fMRI). DTI and rs-fMRI data are not reported here. Research protocol duration was nominally 7.5 min, but imaging times in practice varied from approximately 10–15 min.

**Table 1:** Participant demographics at the time of imaging and subsequent fetal outcome.

Participant	Maternal age at the time of imaging (years)	Gestational age at the time of imaging (weeks)	Fetal outcome
1	37	34 + 1	Normal vaginal delivery at 37 weeks of gestation (WG). 6q27 deletion
2	38	35 + 3	Cesarean delivery at 39 WG for placenta previa. Ongoing postnatal hydrocephalus and ventriculomegaly
3	36	36 + 3	Cesarean delivery at 40 WG for macrocephaly.
4	32	32 + 4	Delivered at other institution—no follow-up
5	37	31 + 5	Delivered at other institution—no follow-up

### 2.4 Image reconstruction and analysis

YB MT images were reconstructed in MATLAB (2023a, The MathWorks Inc., Natick, MA, USA RRID: SCR_001622) from the raw *k*-space data exported from the scanner and the acquisition trajectory file. The reconstruction produced the four images listed in the caption of Figure 3 (PDw, PDw attenuated by SS-driving acquisitions that account for *T*_1_ and *B*_1_ dependence, SS PDw image attenuated by the MT saturation pulse block, and PDw attenuated by the CEST saturation pulse block), alongside *T*_1_–*B*_1_-weighted, MTw, and CESTw images as calculated voxelwise in Equations 1–3 (20, 21).

(1) $$T_{1}-B_{1}-\mathrm{weighted}\;\;=\;\;\frac{T_{1}-B_{1}-\mathrm{attenuated}}{PDw}$$

(2) $$\mathrm{MTw}\;\;=\;\;\frac{\left(T_{1}-B_{1}-\mathrm{attenuated})\;\;-\;\;(MT-\mathrm{attenuated}\right)}{T_{1}-B_{1}-\mathrm{attenuated}}$$

(3) $$\mathrm{CESTw}\;\;=\;\;\frac{\left(\mathrm{MT}-\mathrm{attenuated})\;\;-\;\;(\mathrm{CEST}-\mathrm{attenuated}\right)}{T_{1}-B_{1}-\mathrm{attenuated}}$$

**Figure 5:**
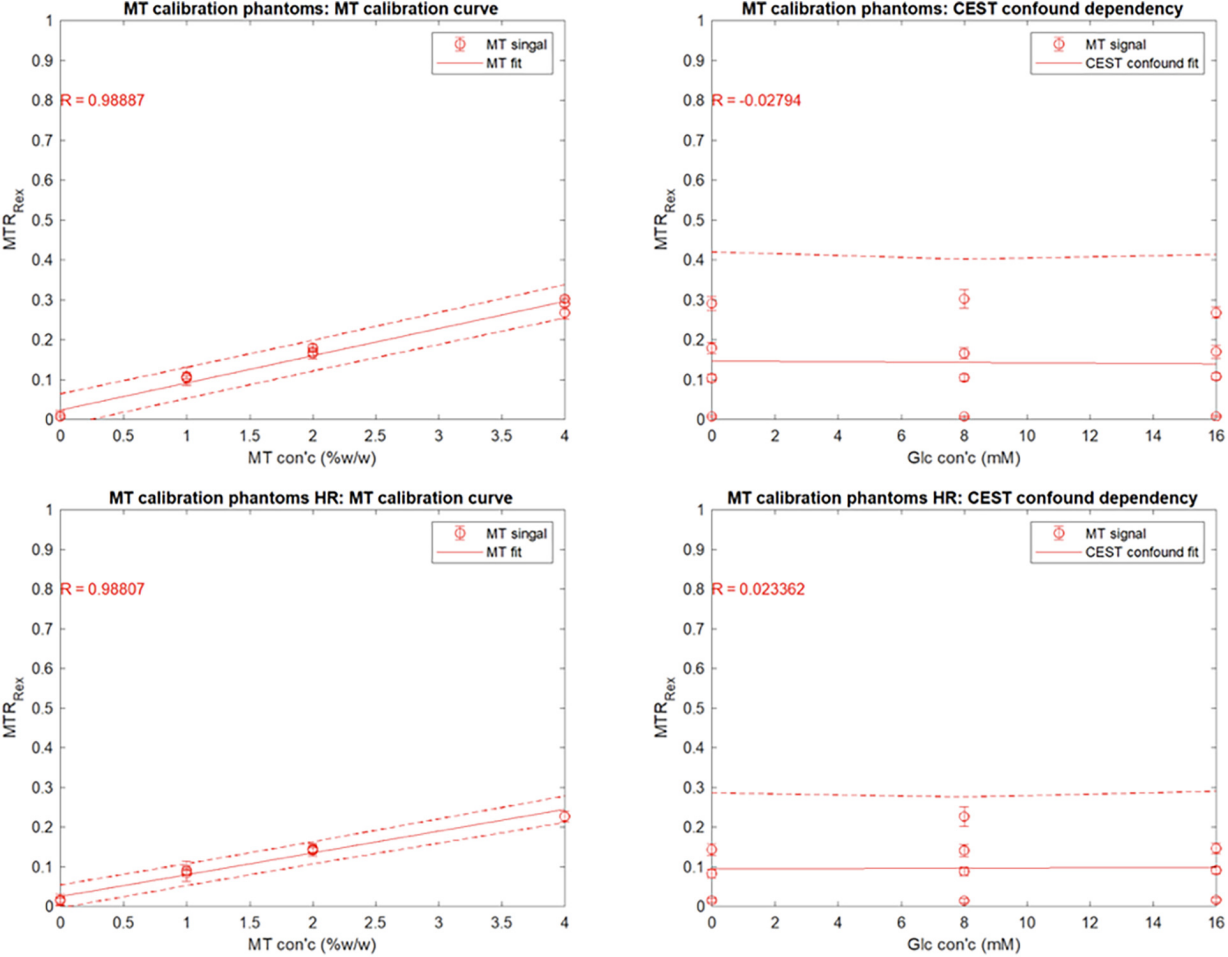
Mean YB MTw image intensity of the ST phantoms using the SR trajectory as a function of **(A)** agar content (% w/w) and **(B)** glucose content (mM). Mean YB MTw image intensity of the ST phantoms using the HR trajectory as a function of **(C)** agar content (% w/w) and **(D)** glucose content (mM).

MTw images of phantoms were used to produce the MT signal and ST confound dependency plots in Figure 5. Regions of interest (ROIs) were determined by binary thresholding of the PDw image using Otsu's method, minimizing interclass variance. This method to mask out the background was not used for participant imaging due to dielectric artefact causing signal dropout (22). To estimate participant motion, the variance of received phase (ϕ) at the centre of *k*-space (*k*_0_) across all interleaves, readouts, and channels was calculated and summarized in Table 2.

**Table 2:** Participant imaging quality assessment, including notable qualitative features and artefacts, and motion estimation quantified through variance of *k*_0_ phase.

Participant	Qualitative image quality	Patient-reported breath hold tolerability	Motion estimation ($\sigma_{\phi (k_{0})}$)
1	Good contrast, minimal spiral blurring, suffers coil sensitivity and dielectric artefacts	Tolerable	1.8280 rad
2	Contrast in prominent features but spiral artefacts and signal pileup reduce contrast and partially occlude fetal brain	Tolerable	1.9138 rad
3	Spiral blurring and motion reduced contrast	Tolerable	1.9074 rad
4	Contrast that aligns with structural features	Tolerable	1.8481 rad
5	Contrast that aligns with structural features	Tolerable	1.9044 rad

## 3 Results

YB MT signal in ST phantoms was linearly correlated with agar concentration, but not with glucose CEST confound concentration, demonstrating sensitivity to semisolid macromolecules and specificity from other saturation transfer confounds (Figure 5). Calibration coefficients were 6.8 ± 0.8 (units of % YB MT signal normalized to PDw signal per %w/w agar) and 5.5 ± 0.7 for 350 mm (SR) and 150 mm (HR) trajectories, respectively, and were significantly different (α=0.05). Limits of detection (LOD) were 0.01%w/w (SR) and 0.69%w/w agar (HR). Limits of quantitation (LOQ) were 0.62%w/w (SR) and 2.74%w/w agar (HR).

**Figure 6:**
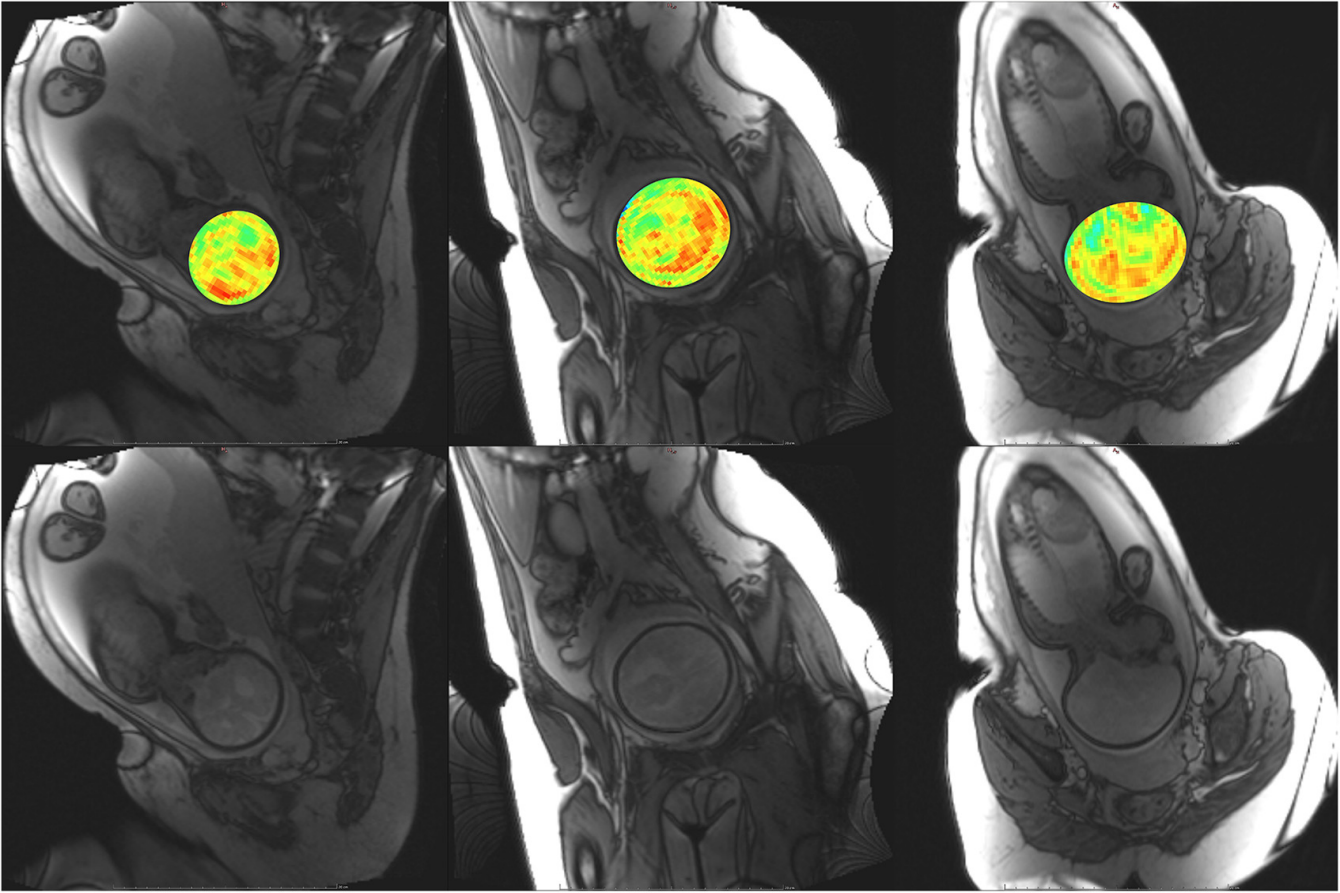
Participant 1. YB MTw image of the fetal brain superimposed on PD/*T*_2_-w three-plane structural image for clarity. Bottom: Corresponding PD/*T*_2_-w three-plane structural images of the fetal brain. Higher MT-weighting is notable for Participant 1 since some saturation bands were closer to on-resonance than for other participants, eliciting a larger MT effect. Ventricles, cerebellum, pons, and brainstem can be appreciated. Enhancement is observed along the PLIC, in regions surrounding the basal ganglia, and along the corpus callosum.

**Figure 7:**
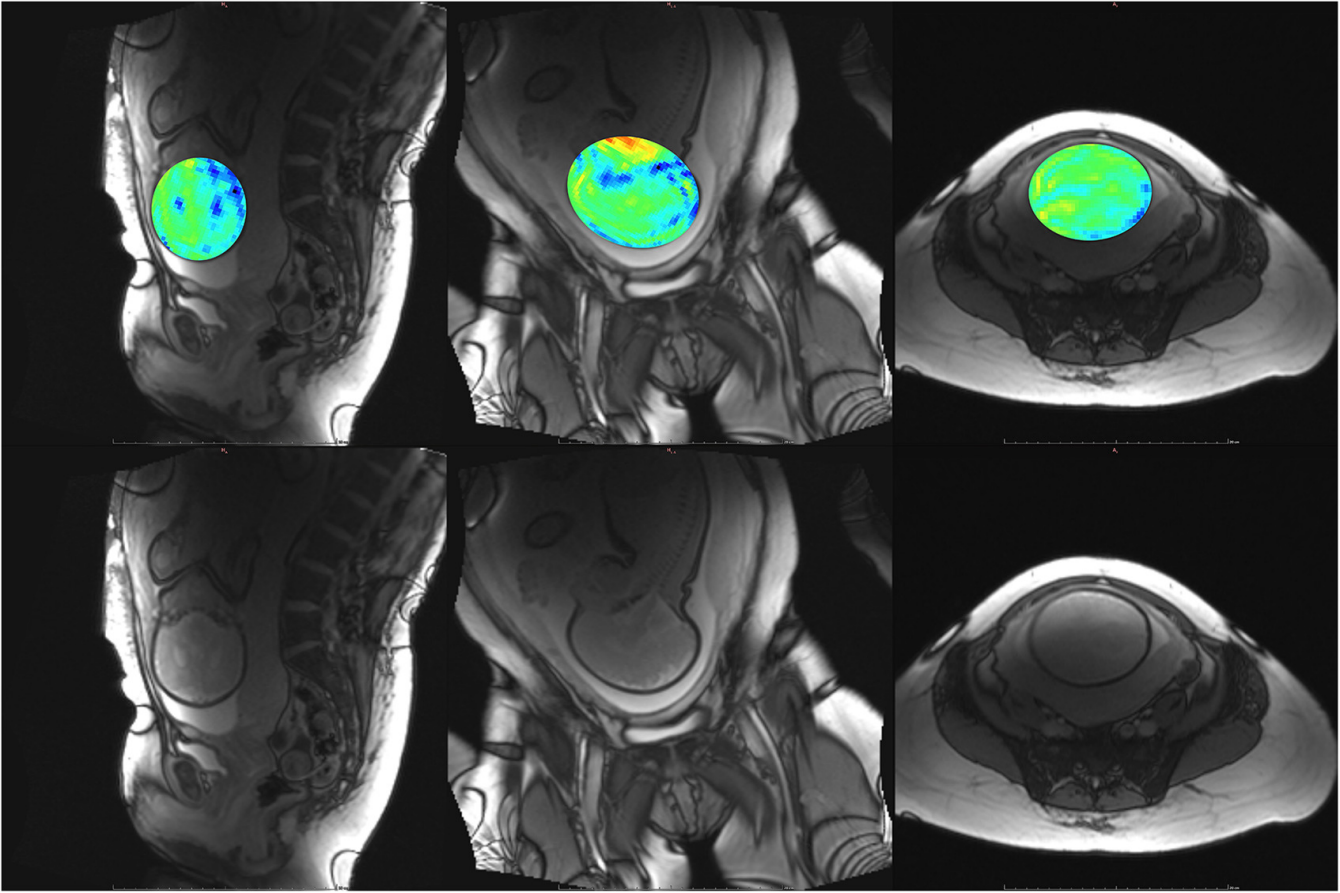
Participant 2. Top: YB MTw image of the fetal brain superimposed on PD/*T*_2_-w three-plane structural image for clarity. Bottom: Corresponding PD/*T*_2_-w three-plane structural images of the fetal brain. The structural image demonstrates dielectric signal dropout, which also appears on YB MT images. This reduces MTw SNR and increases the appearance of spiral artefacts near the fetal brain.

**Figure 8:**
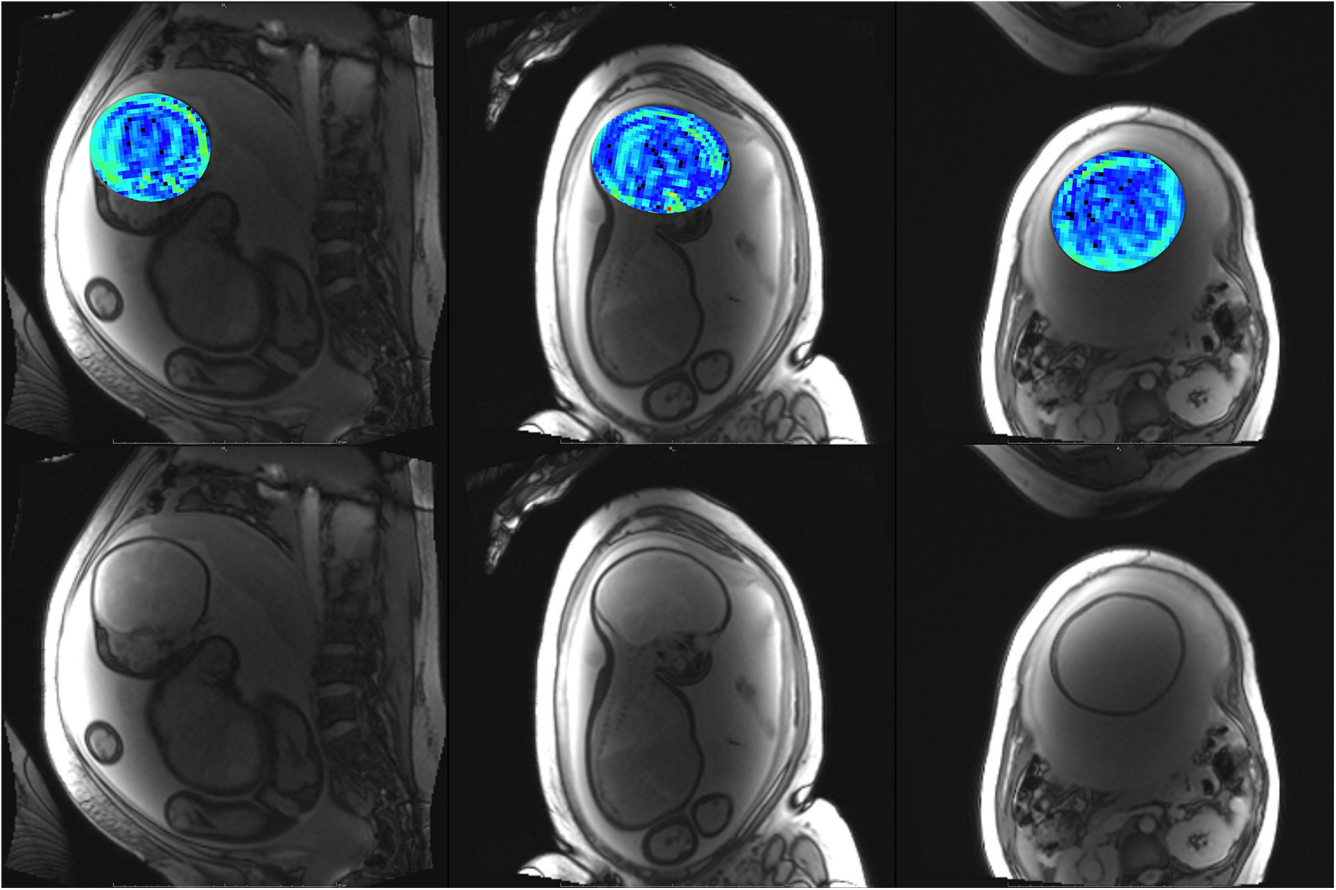
Participant 3. Top: YB MTw image of the fetal brain superimposed on PD/*T*_2_-w three-plane structural image for clarity. Bottom: Corresponding PD/*T*_2_-w three-plane structural images of the fetal brain. These images were acquired after the participant had been imaged on the clinical scanner for 1.5 h, resulting in poor breath hold compliance.

**Figure 9:**
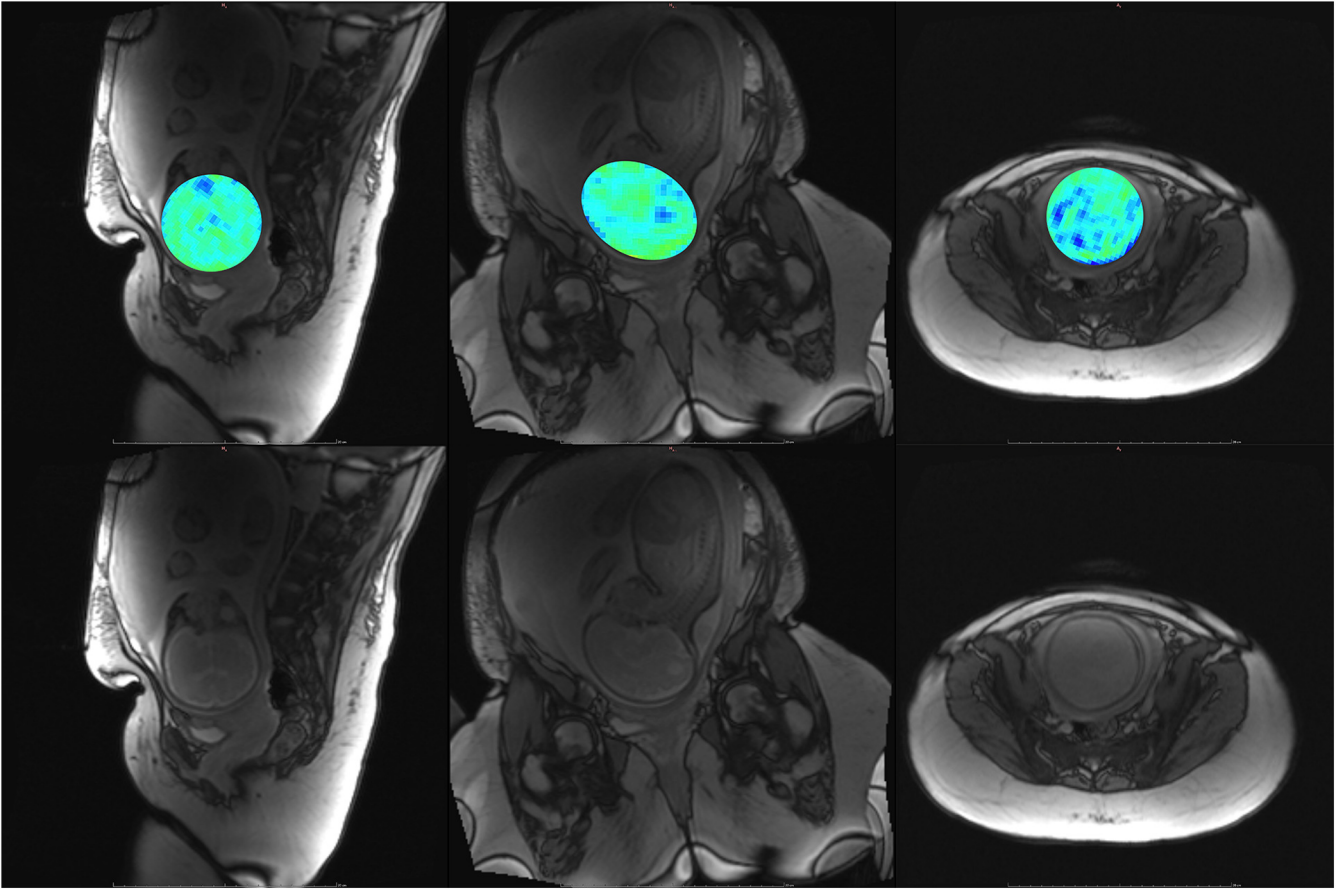
Participant 4. Top: YB MTw image of the fetal brain superimposed on PD/*T*_2_-w three-plane structural image for clarity. Bottom: Corresponding PD/*T*_2_-w three-plane structural images of the fetal brain. Some fetal brain structures can be observed in these images, including the brainstem, ventricles, and lateral sulcus. Participant 4 suffered anxiety and claustrophobia, resulting in low compliance with breath hold instructions.

**Figure 10:**
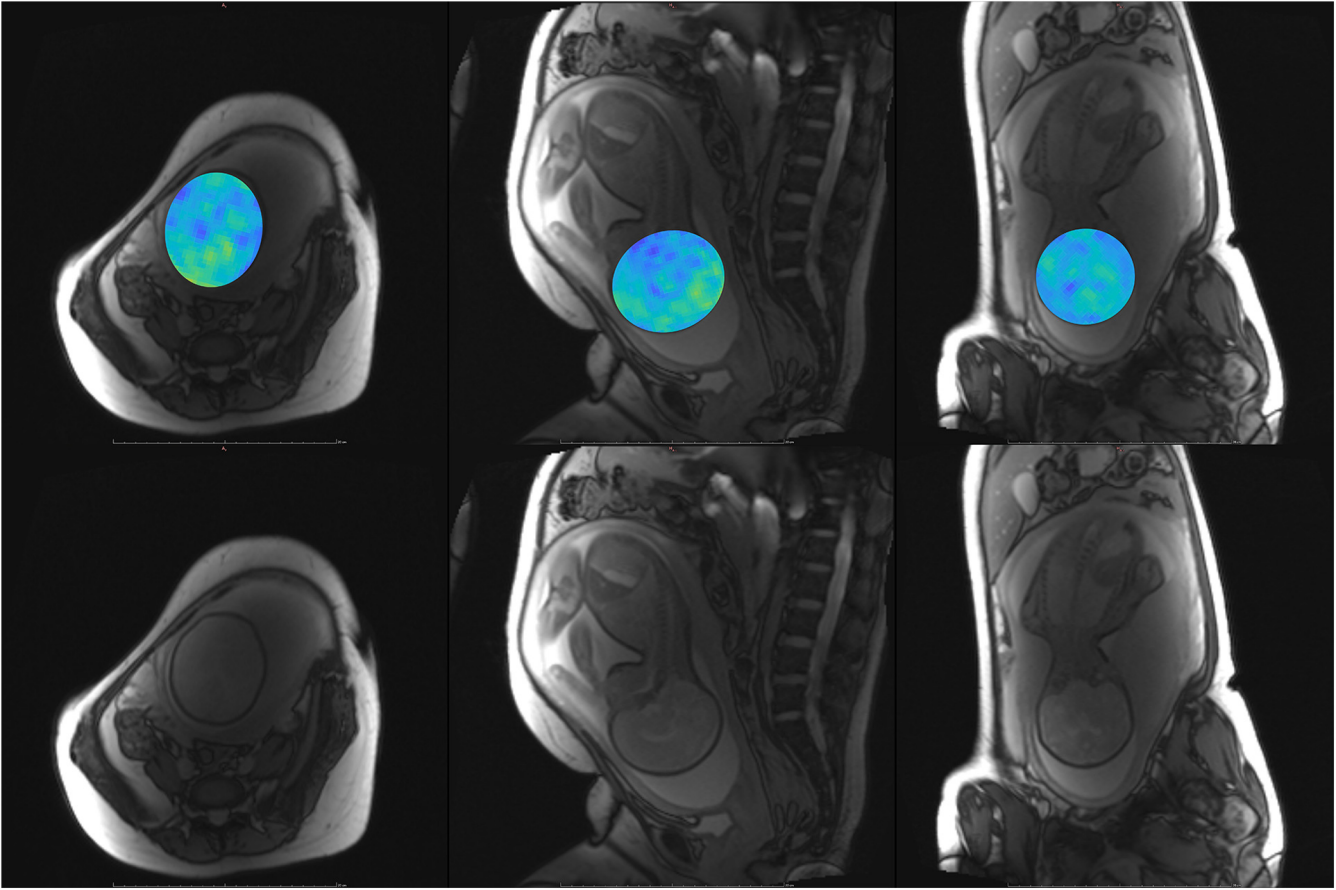
Participant 5. Top: YB MTw image of the fetal brain superimposed on PD/*T*_2_-w three-plane structural image for clarity. Bottom: Corresponding PD/*T*_2_-w three-plane structural images of the fetal brain.

Participant YB MT-derived MTw images have been masked to the fetal brain and superimposed on PD/*T*_2_-w three-plane structural images in Figures 6–10 to allow comparison of features to structural imaging. MTw signal is presented in a jet colourmap with a dynamic range from 0% to 70%. Patient-reported breath hold tolerability and qualitative image quality are summarized in Table 2.

## 4 Discussion

The phantom and *in vivo* image data demonstrate that YB MT is sensitive to physiological MT and specific to MT contrast in the presence of CEST agents that can act as a saturation transfer confound. YB MT additionally produces an image with contrast that follows expected fetal features in the third trimester. *In vivo* imaging further demonstrates that the breath hold duration is manageable and that motion artefacts in gestational imaging can be somewhat mitigated using highly accelerated single breath hold sequences. YB MT image contrast broadly aligns with structural features *in vivo*, with notable hyperintensities immediately proximal to the cortical surface [Figures 6–10 (top)] not seen in structural imaging [Figures 6–10 (bottom)]. Also not seen in structural images, many of the participants' YB MT images show hyperintensities inferior to the lateral ventricles demonstrating enhancement of the posterior limb of the internal capsule (PLIC), at the pons, along the corpus callosum in sagittal planes, and in the subthalamic region.

MT sensitivity in YB MT is limited by the small SSFP flip angle, voxel size, and dielectric signal dropout prevalent at higher field strengths in this anatomy. The Ernst angle for fetal tissues alone is higher than that of the phantoms, so greater sensitivity *in vivo* could be obtained by using a higher FA. The improved MTw contrast for Participant 1 demonstrates SNR improvements possible with only saturation pulse band adjustment. The saturation pulse was modified to obtain amide/amine proton transfer-weighted (APTw) images thereon (21). The smaller saturation band offsets of the earlier saturation pulse used with Participant 1 did, however, result in greater MT sensitivity than the MT-APT saturation pulse and could be revisited in future implementations. While efforts were made to position the 18-channel flex coil proximal to the fetal head, coil sensitivity falloff and signal dropout due to dielectric effects seen in the structural images in Figures 6–10 (bottom) resulted in lower MT SNR and increased appearance of other artefacts, such as spiral blurring particularly apparent in Figures 2 and 3. Fat saturation may be incorporated in the future to reduce off-resonance artefacts contributing to spiral blurring. Variable placement of the flex coil array relative to the spine coil could contribute to signal falloff. The addition of a smaller flexible coil array directly above the fetal head could be investigated in future imaging, with the above caveats. MT sensitivity further proved to limit achievable resolution, as demonstrated with the HR trajectory. While the HR trajectory did allow for phantom imaging, LOD and LOQ were substantially higher than with the SR trajectory. While the LOQ of the HR trajectory is below the expected physiological MT signal, along with the other above limitations to MT sensitivity, the HR trajectory was not translatable to *in vivo* imaging. Future work may focus on improving HR trajectories' SNR by oversampling, interleaving saturation pulses, and better modelling of gradient hysteresis to reduce spiral artefacts.

Despite all participants reporting that the breath hold was tolerable, motion artefacts continued to be a challenge. YB MT was run following both DTI and rs-fMRI sequences that were observed to reliably elicit fetal motion, both during these acquisitions and in subsequent acquisitions. Reconstruction was done offline, preventing the detection of these motion artefacts and subsequent reacquisition at the scanner. Future implementations could include online recon for more seamless translation to clinical use. Adjusting the yarnball trajectory to more densely oversample central *k*-space could benefit motion robustness. Saturation and magnetization recovery times account for 8 s of the breath hold, while acquiring the three breath hold contrasts occupies 5 s; hardware limitations of the Prisma prevent more effective saturation of slow ST such as in MT, limiting the sensitivity improvement and artefact mitigation possible during acquisitions. Migration to scanner hardware capable of CW saturation could overcome some of these challenges and better enable translation to *in vivo* imaging. Longer CW saturation also narrows linewidth, potentially enabling lower-field imaging, which would mitigate dielectric signal dropout. Alternative trajectories to be explored in future work include a stack of spirals, as well as some zero echo time (ZTE) trajectories that are less demanding on gradient performance between TRs. Other hardware improvements, such as parallel transmit (pTx), could also address signal dropout and lack of sensitivity in the centre of the imaging volume.

Participants 1–3 had complicated pregnancies that could have affected the MT contrast, as summarized in Table 1. Participant 3 was unable to remain still through imaging, and so significant motion artefacts in their images overwhelmed MT contrast. At the time of manuscript writing, Participants 4 and 5 have given birth at institutions outside those covered by ethics approval, so fetal outcomes are unknown. Participant 4 suffered severe anxiety related to MRI that presented as patient motion and limited imaging time.

Participants have yet to meet eligibility criteria or complete postnatal follow-up imaging, but recruitment to validate YB MT against a postnatal imaging timepoint is ongoing. The planned postnatal imaging will allow for prenatal YB MT contrast comparison to both postnatal YB MT and with more established MT pulse sequences that have long acquisition times better suited to sleeping babies. *Ex utero* validation was not explored, due to tissue temperature and pH having a large effect on the ST mechanism and contrast. DTI imaging is also being acquired for comparison of YB MT to apparent diffusion coefficient and fractional anisotropy maps as biomarkers of axonal development (23).

The developed YB MT pulse sequence demonstrates sensitivity to physiological MT and acquires within a tolerable breath hold *in vivo*. Phantom imaging indicates the multiband saturation in YB MT is specific to MT signal and with minimal contributions from narrow linewidth confounds like CEST and NOE (24). This selectivity arises from limited coherent irradiation for narrow linewidth analytes, preferentially saturating broad contributions such as symmetric MT. The specificity of this multiband saturation to symmetric MT allows for the isolation of this ST contribution from the *Z*-spectrum, enabling the generalized translation of rapid CEST and asymmetric MT imaging to clinic without the need for whole-*Z*-spectrum sampling or multipool fitting to remove MT as a confound. The YB MTw image from Participant 1 demonstrates resolution and contrast sufficiently to appreciate several fetal brain structures prominent in early myelination, such as PLIC, the region surrounding the basal ganglia, and the corpus callosum as shown in Figure 6. Together, these results indicate that the sequence could be viably translated to MT imaging of the fetal brain and is a step towards clinical translation of fetal myelin imaging.

## Data Availability

The raw data supporting the conclusions of this article will be made available by the authors, without undue reservation.
